# Perceptions and Experiences of Healthcare Providers and Patients Towards Digital Health Services in Primary Health Care: A Cross-Sectional Study

**DOI:** 10.7759/cureus.58876

**Published:** 2024-04-23

**Authors:** Kumaragurubaran P, Trupti Bodhare, Samir Bele, Vijaya Ramanathan, Thendral Muthiah, Gavin Francis, Ramji M

**Affiliations:** 1 Directorate of Public Health & Preventive Medicine, Government of Tamil Nadu, District Health Office, Madurai, IND; 2 Community & Family Medicine, All India Institute of Medical Sciences, Madurai, Madurai, IND; 3 Community Medicine, Velammal Medical College Hospital and Research Institute, Madurai, IND; 4 Anatomy, All India Institute of Medical Sciences, Madurai, Madurai, IND

**Keywords:** primary health care, digital health, mhealth, patients, women health volunteers, healthcare providers

## Abstract

Background: Digital health has the potential to help achieve the Sustainable Development Goals (SDGs) by supporting health systems and enhancing health promotion and disease prevention. However, obstacles such as restricted internet access, inadequate technical assistance, clinical resource disparity, and insufficient user training can impede the utilization and growth of digital health. Researchers should examine healthcare providers' and patients' perspectives to identify challenges and enhance usability.

Methodology: The study was conducted among women health volunteers, staff nurses, and patients who used the VinCense mobile application (MedIoTek Health Systems Private Limited, Chennai, India) to record vital signs. A semi-structured questionnaire was used to evaluate participants' sociodemographic characteristics, perception of digital health monitoring, and patients' attitudes toward digital health monitoring devices. The data were analyzed using R programming, Version 4.3.3 (www.r-project.org). A multinomial logistic regression analysis was used to examine the association between sociodemographic characteristics and attitudes of patients toward digital health monitoring.

Results: The study involved 27 healthcare providers and 406 patients. The majority (66.6%) of healthcare providers found the device convenient and efficient. Around 74.1% faced technical difficulties like internet connectivity and device battery issues. Among patients, 79.8% were satisfied with their digital health monitoring experience, 86.2% found device usage comfortable and 78.1% expressed satisfaction with health education and feedback. Around 354 (87.2%) patients stated that technology has improved healthcare, and 326 (80.3%) said that health technologies have improved ease. The results indicate that female gender (p=0.00), age above 50 years (p=0.04), and occupation status as a semiskilled worker (p=0.03), skilled worker (p=0.00), and clerical/shop/farmer (p=0.01) were statistically significant and associated with the positive attitude for digital health monitoring.

Conclusions: The digital health monitoring experience was found satisfactory by both patients and healthcare providers. The mobile health (mHealth) has tremendous potential for enhancing patient health. Therefore, it is advisable to contemplate an expansion of the VinCense mHealth Platform and other digital solutions to improve service delivery in primary healthcare setups.

## Introduction

India's public health system faces multiple challenges. These challenges include inadequate infrastructure and manpower, urban-rural inequities, restricted health insurance coverage, and insufficient public healthcare budget. Non-communicable diseases (NCD) and maternal and child mortality are threatening India's healthcare system [1]. The country has experienced a rise in non-communicable diseases over the past decade, attributing to an estimated 4.7 million deaths accounting for 49% of all-cause mortality [2].

On comparing the data sets of the National Family Health Survey (NFHS-4), the NFHS-5 shows a dramatic rise in the prevalence of hypertension among men and women, increasing from 15% and 11% to 24% and 21%, respectively. In the same way, the percentage of women who reported blood glucose levels above 140 mg/dl has risen from 5.8% to 12%. These illnesses result in a substantial demand for healthcare services and can be expensive, especially in regions with scarce resources. Additionally, the findings demonstrate the importance of reducing newborn and child mortality by strengthening primary health care [3].

The field of digital health is expanding rapidly revolutionizing the health systems and healthcare delivery worldwide. Digital health encompasses various terms such as telemedicine, electronic health (e-health), mHealth, and digital informatics. To meet the sustainable development goals (SDGs), notably SDG 3, United Nations agencies, member states, and diverse organizations are integrating technology into healthcare systems to improve service delivery globally, eventually and to achieve universal health coverage. Digital health has great potential to help achieve the SDGs by supporting health systems and enhancing health promotion and disease prevention [4]. The convergence of medicine and technology can leverage advanced and affordable platforms in developing nations to improve global and local capacities and develop a specific level of self-reliance. Enhancing public health services in less developed countries is significantly improved by mHealth/eHealth, especially in areas with limited clinical resources and healthcare facilities. In such locations, technology supports treatment and prevention. Various technologies are available to provide health information that can enhance the health literacy of patients and healthcare workers [5].

Aligned with the Make in India initiative and Digital India program, the Ministry of Health & Family Welfare launched governance initiatives with the ambitious aim of digitizing national healthcare sectors to enhance access to affordable and standardized healthcare services [6].

The Department of Public Health and Preventive Medicine, Deputy Director of Health Services Madurai, is utilizing the VinCense Digital Health Screening Platform (DHSP)/Spot Check (MedIoTek Health Systems Private Limited, Chennai, India) to record vital signs such as pulse rate, oxygen saturation, blood pressure, and blood glucose levels of patients in Zone 2 Urban Primary Health Centres (UPHCs) under the Makkalai Thedi Maruthuvam Scheme [7].

The use of mHealth technology has been proposed as a way to alleviate the strains that various diseases impose on healthcare systems. Standardizing procedures and simplifying diagnostic and monitoring equipment for mobile devices like phones and tablets can improve the efficiency and service range of doctors and paramedical staff [8]. There has been a remarkable rise in mobile phone and internet users in recent years in developing countries due to a significant drop in the cost of these devices and their connectivity services. Several mobile health applications with structured programs are being provided to community health workers to help improve the management of diseases in communities [9].

Notwithstanding these benefits, obstacles such as restricted internet access, inadequate technical assistance, clinical resource disparity, and insufficient user training can impede the utilization and growth of mHealth. To fully leverage the advantages of mobile technologies for healthcare workers policymakers, program administrators, and researchers must comprehend the specific circumstances and constraints associated with these tools [10].

Researchers should examine healthcare providers' and patients' perspectives to identify the challenges and enhance usability. However, there is a scarcity of research that examines patients' and healthcare providers' perspectives on health applications (apps) and the difficulties they face, as well as the characteristics linked to low satisfaction with health apps. [11]

We hypothesized that various factors, such as sociodemographic characteristics and prior experience with digital health services, determine the perceptions of healthcare providers and patients. These factors serve as precursors to their overall attitude. The device's ease of use and minimal technical issues will result in a positive attitude and acceptance of the app. We conducted this cross-sectional study over three months among patients and healthcare providers to explore their perceptions and experiences regarding the acceptability, benefits, and challenges of using the Vincense Spot Check for monitoring vitals. In addition, the study intended to analyze the patients' perspectives on telemedicine and its correlation with social and demographic factors. This study would be highly advantageous as the findings could assist in formulating guidelines for the future advancement of health applications, the technological intricacy and simplicity of applications based on demographic data, and other factors explored in this study. The government and technology enterprises might use the results to develop a user-friendly health application that enhances the involvement of both patients and healthcare providers with the app.

## Materials and methods

Study design and setting

This is a community-based cross-sectional study undertaken in Zone 2 of Madurai District, comprising eight Urban Primary Health Centres (UPHCs) where the Vincense Spot Check tool is being used for digital health recording. The research was conducted over three months, spanning from October 2023 to December 2023.

Participants

The study participants included women health volunteers (WHVs), staff nurses, and patients who used the VinCense app to record blood pressure, pulse rate, oxygen saturation, and blood glucose.

Inclusion and exclusion criteria

The research comprised individuals who used the VinCense Spot Check for digital monitoring and gave written informed consent. Study participants who did not utilize the VinCense Spot Check or declined participation were excluded.

VinCense Spot Check tool

**Figure 1:**
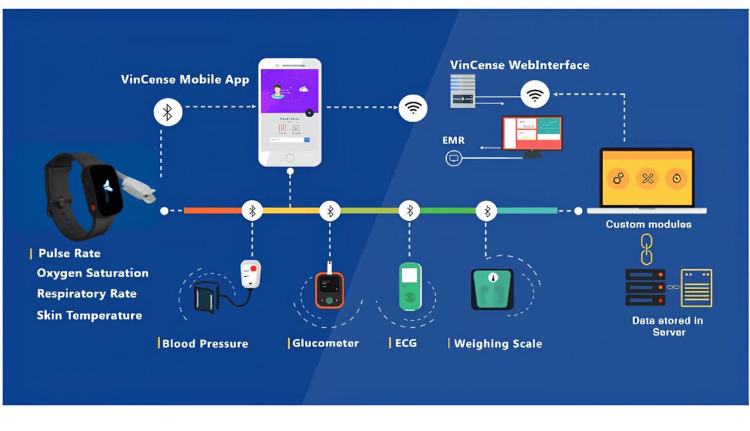
The Vincense Spot Check platform *Image Credit: MedIoTek Health Systems Private Limited, Chennai, India

VinCense Digital Health Screening Platform (DHSP)/Spot Check, developed by MedIoTek Health Systems Private Limited, Chennai, India can provide eight parameters in four minutes for NCD screening. This healthcare tool is designed to record vital signs such as pulse rate, oxygen saturation, blood pressure, and blood glucose levels of patients (Figure 1). The VinCense mHealth platform measures the following parameters using the Bluetooth smart-enabled VinCense wearable, non-invasive blood pressure monitor, glucometer, and digital weighing scales and pushes the data to the cloud in real time through the VinCense mobile app. The NCD analytics in the web interface can identify early NCD metabolic risk factors like elevated blood pressure, blood sugar, and obesity from the collected vitals.

Variables and data measurement

Researchers used a semi-structured questionnaire to evaluate the socioeconomic background of the participants. The socioeconomic status of the participants was evaluated using an updated version of the modified Kuppuswamy socioeconomic scale for the year 2023. A score between 26 and 29 indicates an upper class; a score of 16 to 25 suggests an upper middle class; a score of 11 to 15 indicates a lower middle class; and a score of 5 to 10 indicates an upper lower class. Finally, a score below 5 signifies a lower class [12]. The perception of the digital monitoring of health status was evaluated in the domain of overall experience, benefits and concerns, comfort and trust, communication and information, and future preferences. Additionally, patients' attitude towards the use of digital health monitoring devices was assessed. The replies were recorded using a five-point Likert scale. A score of 1 indicates a severe disagreement, a score of 2 indicates a disagreement, a score of 3 indicates neutrality, a score of 4 indicates agreement and a score of 5 indicates a strong agreement. Typically, lower ratings suggest disagreement or dissatisfaction whereas higher levels imply stronger agreement or greater satisfaction. The questionnaire was developed based on a literature review and expert consultation. The questionnaire's reliability was evaluated using Cronbach's alpha, resulting in a value over 0.7.

Study size and sampling

The study included participants from all eight urban primary care centers in Zone 2 of the Madurai district. The Vincense Spot Check is being used for digital health recording in Zone 2 of the Madurai district; hence, it was selected using convenient sampling. The patients and all healthcare providers, including WHVs and staff nurses working in the UPHC and involved in digital monitoring were approached and included in the study through convenient sampling. Based on the findings from the previous study, the sample size for patients was calculated to be 400 using the formula n = [Z^2^ (P)(1-P)]/ d^2^, with a 95% level of significance and a 5% margin of error. The prevalence of 56.0% was considered [13]. The resulting value of n was 358, which was rounded up to 400. Therefore, a minimum sample size of 400 was required to conduct the study.

Pilot study

A pilot study with 20 participants assessed technical feasibility and ensured proper question sequence and presentation. The pilot study participants were different from the primary study sample, ensuring that any adjustments made, based on the pilot findings, did not affect the main study results.

Ethical considerations

Before commencing the research, approval from the Institutional Ethics Committee of Velammal Medical College Hospital & Research Institute Madurai (VMCIEC/092/2023) was obtained. The researchers have provided comprehensive information about the study and clarified that participation is voluntary, with the ability to withdraw without facing any consequences. Written informed consent was obtained from study participants. Ensuring the utmost security, all participant identifying data is securely locked and replaced with code numbers. The data is protected with passwords to maintain confidentiality.

Statistical analysis

The data were analyzed using R programming, Version 4.3.3 (www.r-project.org). The baseline characteristics, attitudes, and experiences of the participants were presented as frequency, percentages, mean, and standard deviation values. A multinomial logistic regression analysis was used to explore the association between the sociodemographic characteristics of patients and with the attitude of digital health monitoring. The attitude mean value is considered an independent variable. The dependent variables in this study consisted of categorical variables, including age, gender, education, occupation, and socioeconomic status. The criteria for statistical significance was a P-value of ≤0.05.

## Results

A total of 27 healthcare providers and 406 patients participated in the study. Table 1 shows the baseline socio-demographic characteristics of healthcare providers and patients. The healthcare providers had a mean age of 35.96 ± 5.82 with the majority having less than five years of experience (85.2%) and 22.2% holding a diploma/degree. The patients in this study had a mean age of 40.46 ± 17.09, with a larger proportion of females (81.3%). Around 30.0% of patients were educated up to a diploma/degree, 92.9% were married, and 71.1% were unemployed. Around 79.6% of patients belong to the socio-economic status of the upper lower class.

**Table 1:** Socio-demographic characteristics of healthcare providers and patients

Variables	Socio- demographic characteristics		Frequency (%)
Healthcare Providers	Age (Mean ± SD)		35.96 ± 5.82
	Experience	Below 5 years	23 (85.2%)
		5 to 10 years	2 (7.4%)
		Above 10 years	2 (7.4%)
	Education	School education	21 (77.8%)
		Diploma/Degree	6 (22.2%)
Patients	Age (Mean ± SD)		40.46 ± 17.09
	Gender	Male	76 (18.7%)
		Female	330 (81.3%)
	Education	Illiterate	23 (5.7%)
		School Education	256 (63.1%)
		Diploma/Degree	122 (30.0%)
		Professional Degree	5 (1.2%)
	Marital Status	Married	377 (92.9%)
		Unmarried	10 (2.5%)
		Divorced	2 (0.5%)
		Widowed	17 (4.2%)
	Occupational status	Unemployed	291 (71.1%)
		Unskilled worker	28 (6.9%)
		Semiskilled work	46 (11.3%)
		Skilled worker	18 (4.4%)
		Clerical/Shop/Farmer	13 (3.2%)
		Semi profession	10 (2.5%)
	Socioeconomic Status	Upper Middle	13 (3.2%)
		Lower Middle	21 (5.2%)
		Upper Lower	323 (79.6%)
		Lower	49 (12.1%)

The majority of 22 (81.5%) of the healthcare providers were using digital health monitoring devices daily in their routine work. Around 24 (88.9 %) of healthcare providers stated that the training provided was effective. Around 18 (66.6%) participants said that it was convenient to use the device and were satisfied with the usability and efficiency of the app. A total of 20 (74.1%) of the healthcare providers had experienced technical problems. The most common technical difficulties were internet connectivity and the battery of the device. The highest mean score (4.74 ± 0.526) was obtained in the domain of support from the technical team in which the majority (77.8%) of the healthcare providers said that the support team was very responsive. Around 23 (85.1%) of healthcare providers said that patients were comfortable and 20 (74 %) said that patients were cooperative while using the device (Table 2).

**Table 2:** Perceptions and experiences of healthcare providers regarding digital health services

Variables	Frequency (%)					Mean ± SD
Effectiveness of training	Highly In-effective	Not Effective	Neutral	Effective	Highly Effective	4.15 ± 0.82
	0 (0%)	2 (7.4%)	1 (3.7%)	15 (55.6 %)	9 (33.3 %)	
Convenience in using the device	Very Difficult	Difficult	Neutral	Easy	Very Easy	3.70 ± 1.203
	3 (11.1%)	0 (0 %)	6 (22.2%)	11 (40.7%)	7 (25.9%)	
Usability and efficiency	Very Dissatisfied	Dissatisfied	Neutral	Satisfied	Very Satisfied	3.85 ± 0.907
	0 (0%)	2 (7.4%)	7 (25.9%)	11 (40.7 %)	7 (25.9 %)	
Technical issues or malfunction	Often	Always	Sometimes	Rarely	Never	3.15 ± 0.818
	0 (0%)	5 (18.5%)	15 (55.6%)	5 (18.5%)	2 (7.4%)	
Support from the technical team	Very Unresponsive	Unresponsive	Neutral	Responsive	Very Responsive	4.74 ± 0.526
	0 (0 %)	0 (0 %)	1 (3.7%)	5 (18.5 %)	21 (77.8%)	
Patients comfort	Very Uncomfortable	Somewhat Uncomfortable	Neutral	Somewhat Comfortable	Very Comfortable	4.19 ± 1.001
	1 (3.7%)	1 (3.7%)	2 (7.4%)	11 (40.7%)	12 (44.4%)	
Patients Cooperation	Very Uncooperative	Somewhat Uncooperative	Neutral	Cooperative	Very Cooperative	4.00 ± 1.00
	0 (0 %)	3 (11.1%)	4 (14.8 %)	10 (37.0%)	10 (37.0 %)	

Table 3 presents the perspectives and experiences of patients regarding digital health services. Overall, 324 (79.8%) patients were satisfied, with their digital health monitoring experience. Around 350 (86.2%) patients considered device usage comfortable, and 317 individuals (78.1%) expressed satisfaction with the information and health education they received from healthcare providers. When questioned about the potential advantages, 167 individuals (41.1%) expressed that it has enhanced the level of convenience, while 134 people (33 %) mentioned that it results in improved communication with healthcare providers. Around 332 (81.8%) patients gave their preference for continuing the use of the app in the future.

**Table 3:** Perceptions and experiences of patients regarding digital health services

Variables		Frequency (%)	Mean ± SD
Overall Experience with Digital Monitoring	Very Dissatisfied (1)	0 (0%)	4.01 ± 0.752
	Dissatisfied (2)	15 (3.7%)	
	Neutral (3)	67 (16.5%)	
	Satisfied (4)	222 (54.7%)	
	Very Satisfied (5)	102 (25.1%)	
Level of comfort while using the device	Very uncomfortable (1)	0 (0%)	4.13 ± 0.685
	Uncomfortable (2)	8 (2.0%)	
	Neutral (3)	48 (11.8%)	
	Comfortable (4)	233 (57.4%)	
	Very comfortable (5)	117 (28.8%)	
Level of information /health education received	Very Dissatisfied (1)	0 (0%)	3.99 ± 0.718
	Dissatisfied (2)	9 (2.2%)	
	Neutral (3)	80 (19.7%)	
	Satisfied (4)	224 (55.2%)	
	Very Satisfied (5)	93 (22.9%)	

The results presented in Table 4 outline patient's attitudes toward the use of technology in the health sector. Around 354 (87.2%) patients stated that technology has improved healthcare, and 326 (80.3%) said that health technologies are easy to use. While 270 (66.5%) patients express concerns about over reliance on technology by doctors and hospitals, the majority 297 (73.1%) agree that technology cannot replace real healthcare providers. Around 308 (75.9%) patients also expressed a desire for increased use of technology in healthcare and 283 (69.7%) patients acknowledged the role of health technology in reducing human error. Furthermore, 310 (76.4%) patients find new developments in health technology exciting, and 308 (75.8%) patients believe that health technology benefits everyone. However, a significant concern arises regarding the privacy of health records, with 70 (17.3%) patients worrying about the security of their data.

**Table 4:** Patients' attitudes towards digital health services

Variables		Frequency (%)	Mean ± SD
Technology has improved healthcare	Strongly Disagree (1)	0 (0%)	4.20± 0.65
	Disagree (2)	0 (0%)	
	Neutral (3)	52 (12.8%)	
	Agree (4)	221 (54.4%)	
	Strongly Agree (5)	133 (32.8%)	
Health technologies are easy to use	Strongly Disagree (1)	0 (0%)	4.14± 0.72
	Disagree (2)	0 (0%)	
	Neutral (3)	80 (19.7%)	
	Agree (4)	188 (46.3%)	
	Strongly Agree (5)	138 (34.0%)	
Healthcare providers rely too much on technology	Strongly Disagree (1)	0 (0%)	3.88± 0.74
	Disagree (2)	0 (0%)	
	Neutral (3)	136 (33.4%)	
	Agree (4)	181 (44.6%)	
	Strongly Agree (5)	89 (21.9%)	
Real doctors and nurses cannot be replaced by technology	Strongly Disagree (1)	0 (0%)	4.05±0.76
	Disagree (2)	0 (0%)	
	Neutral (3)	109 (26.8%)	
	Agree (4)	169 (41.6%)	
	Strongly Agree (5)	128 (31.5%)	
Advocate for further integration of technology in the healthcare sector	Strongly Disagree (1)	0 (0%)	4.07± 0.74
	Disagree (2)	0 (0%)	
	Neutral (3)	98 (24.1%)	
	Agree (4)	183 (45.1%)	
	Strongly Agree (5)	125 (30.8%)	
Health technology reduces human error	Strongly Disagree (1)	0 (0%)	3.94± 0.74
	Disagree (2)	0 (0%)	
	Neutral (3)	123 (30.3%)	
	Agree (4)	185 (45.6%)	
	Strongly Agree (5)	98 (24.1%)	
The prospect of advancements in health technology is exciting	Strongly Disagree (1)	0 (0%)	4.08± 0.74
	Disagree (2)	0 (0%)	
	Neutral (3)	96 (23.6%)	
	Agree (4)	183 (45.1%)	
	Strongly Agree (5)	127 (31.3%)	
Health technology is beneficial for everyone	Strongly Disagree (1)	0 (0%)	4.08± 0.75
	Disagree (2)	0 (0%)	
	Neutral (3)	98 (24.1%)	
	Agree (4)	178 (43.8%)	
	Strongly Agree (5)	130 (32%)	
Concern that technology will not keep my health records private	Strongly Disagree (1)	95 (23.4%)	3.48±1.17
	Disagree (2)	105 (25.9%)	
	Neutral (3)	136 (33.5%)	
	Agree (4)	40 (9.9%)	
	Strongly Agree (5)	30 (7.4%)	

A multinomial logistic regression presented in Table 5 reveals the association between baseline sociodemographic characteristics of patients with the mean attitude score (3.59 ± 0.42) of digital health monitoring. The results indicate that female gender (p=0.00), age above 50 years (p=0.04), and occupation status as a semiskilled worker (p=0.03), skilled worker (p=0.00), and clerical/shop/farmer (p=0.01) were statistically significant and associated with the positive attitude for digital health monitoring.

**Table 5:** Socio-demographic correlates of patients' attitudes toward digital health services *Statistically significant (p≤0.05), ** Reference category

Variable		Coefficient	Standard Error	Significance
Gender	Male	^**^REF	^**^REF	^**^REF
	Female	1.04	0.26	*0.00
Age	Below 30	^**^REF	^**^REF	^**^REF
	31 to 50	-0.34	0.25	0.52
	Above 50	0.19	0.23	*0.04
Education	Illiterate	^**^REF	^**^REF	^**^REF
	School Education	-0.66	0.44	0.14
	Degree/Diploma	-0.68	0.45	0.28
	Professional Degree	-1.30	0.99	0.15
Occupational Status	Unemployed	^**^REF	^**^REF	^**^REF
	Unskilled worker	-0.10	0.39	0.79
	Semiskilled worker	-0.67	0.31	*0.03
	Skilled worker	-1.72	0.58	*0.00
	Clerical/Shop/Farmer	1.68	0.67	*0.01
	Semi-professional	-1.17	0.71	0.09
Socio-economic Status	Upper Middle	^**^REF	^**^REF	^**^REF
	Lower Middle	0.14	0.69	0.84
	Upper lower	0.20	0.55	0.71
	Lower class	-0.23	0.61	0.71

## Discussion

The present study aimed to assess the perspectives of primary healthcare providers and patients regarding the practicality, acceptability, benefits, and obstacles associated with utilizing a mobile application-based device for the monitoring of vitals like pulse rate, oxygen saturation, blood pressure, and blood glucose levels of patients in primary healthcare settings in Madurai district, Tamil Nadu, India. Overall, the study found that a majority of healthcare providers, as well as patients, had a strong interest, positive attitude, and willingness to use mobile app-based devices to assist in the management of disease.

This study revealed that a significant number of healthcare providers expressed their satisfaction with the convenience, usability, and efficiency of the device and app. Our findings are consistent with those of other studies [14,15]. Specialized software can greatly improve the efficiency of healthcare provider's workflow. It enables seamless documentation of services, facilitates information sharing, and ultimately generates the necessary data required for the health system.

The majority of healthcare providers claimed they faced technical difficulties with the equipment. This is similar to the studies conducted by different authors who utilized both qualitative and quantitative methods. These studies revealed common themes such as security and privacy concerns, connectivity, and speed issues with devices commonly reported by health workers [16,17]. Ensuring prompt assistance to healthcare providers is paramount, to optimize efficiency and get precise outcomes. We obtained the highest score in the domain of support from the technical team. A considerable proportion (77.8%) of healthcare providers praised the support team's exceptional responsiveness.

Overall, 324 (79.8%) patients were satisfied, with their digital health monitoring experience. Around 350 (86.2%) patients considered device usage comfortable, and 317 individuals (78.1%) expressed satisfaction with the information, health education, and feedback they received from healthcare providers. Study results showed that device use has increased convenience for 41.1% of patients and improved communication with healthcare providers for 33% of patients. A study conducted by Bhandari et al. showed that patients had the perception that the mHealth method would be beneficial in terms of enhancing medication adherence and promoting behavioural modifications and highly valued the reliability of the information provided by health authorities. Bauer et al. found that more patients felt comfortable sharing mobile health information with clinicians, and health technology may offer scalable health education and self-management assistance, especially for patients [18,19].

The majority (81.8%) of patients preferred continuing the use of the app in the future. The continued use of mHealth apps relies on users evaluating the app's capabilities and their dedication to achieving their health goals. Mallenius et al. found that older patients were interested in adopting mobile phones and services, but they desired actual value [20].

In general, the evidence indicates a favourable attitude towards digital health monitoring devices, however, privacy concerns were present. The majority (80.3%) of patients stated that health technologies have increased the ease of use. Around two-thirds (75.9%) of patients advocated more use of technology in healthcare and felt that health technology benefits everyone. However, around 17.3% of patients were worried about health record privacy. The findings of our study are consistent with the results reported by Jenkins et al. [21] and McGillicuddy et al. [22], who reported positive attitudes among the patients. Jenkins et al. showed that 85% were comfortable with a healthcare professional using mHealth technologies to monitor personal health information, 78.3% believed it would remind them to follow doctor instructions, and 83.3% were confident it could be used to communicate with healthcare providers. Similarly, the findings reported by McGillicuddy et al. indicate that 80% were comfortable with remote health monitoring, 76% were satisfied that their privacy would be sufficiently safeguarded and 87% said monitoring technologies would enhance communication with healthcare professionals regarding medical issues [21,22].

There was a statistically significant association between the favourable attitude toward digital health monitoring and factors such as female gender (p=0.00), age above 50 years (p=0.05), and skilled profession (p=0.00). Our findings are consistent with Jenkins et al. who reported that women were more likely than males to strongly agree with their willingness to adopt mHealth with continuing technical assistance and believed that mHealth may enhance provider communication (p=0.024 and p=0.047). Age also affected the desire to use mHealth. With each year of age, the likelihood of strongly agreeing increased by 4% (odds ratio (OR)=1.04; p=0.046) [21].

Limitations

The participants were recruited from eight UPHCs of the Madurai district, which raises concerns about the generalizability of the findings. Furthermore, those who choose to participate in the study may possess a preexisting inclination towards a favourable disposition towards mHealth, thereby introducing a bias.

## Conclusions

The digital health monitoring experience was found to be satisfactory by both patients and healthcare providers. Healthcare providers conveyed their contentment with the device and app's convenience, usability, and efficiency. Most healthcare providers reported technical concerns like internet connectivity and battery life and commended the support team's response. Patients found the device usage to be convenient and comfortable and were satisfied with the health education, and feedback provided by healthcare providers. They also expressed a preference for continuing to use the app in the future. Overall, patients had a positive attitude toward digital health monitoring devices however, privacy concerns were evident.

The mHealth has tremendous potential for enhancing patient health, particularly in the prevention and management of chronic illnesses, with a focus on personalized care. Future research could focus on measurable health impacts to strengthen the case for mHealth interventions. It is advisable to contemplate an expansion of the mHealth Platform like VinCense or other digital solutions to improve service delivery in primary healthcare setups.

## Appendices

                                                                                              Questionnaire

Perceptions and Experiences of Healthcare Providers and Patients towards Digital Health Services in Primary Health Care

Part A: Healthcare provider’s perceptions towards digital health services

Section 1: Socio-demographic characteristics of healthcare providers

1.1  Age in years:

1.2  Gender:

1.3 Educational status:

1.4 Healthcare facility/institution currently working:

1.5 Duration of working in a healthcare facility: 

Section 2: Perceptions and experiences of healthcare providers regarding digital health services

2.1 How effective was the training provided for using the new device?

o   Highly Ineffective

o   Not Effective

o   Neutral

o   Effective

o   Highly Effective

2.2 How convenient do you find using the new device on your patients?

o   Very Difficult

o   Difficult

o   Neutral

o   Easy

o   Very Easy

2.3 How satisfied are you with the usability and efficiency of the new device?

o   Very Dissatisfied

o   Dissatisfied

o   Neutral

o   Satisfied

o   Very Satisfied

2.4 How often you faced technical issues or malfunctions with the device?

o   Often

o   Always

o   Sometimes

o   Rarely

o   Never

2.5 How responsive is the technical support team when you encounter issues with the device?

o   Very Unresponsive

o   Unresponsive

o   Neutral

o   Responsive

o   Very Responsive

2.6 How comfortable do your patients generally feel when digital health monitoring devices are used on them?

o   Very Uncomfortable

o   Somewhat Uncomfortable

o   Neutral

o   Somewhat Comfortable

o   Very Comfortable

2.7 Do patients generally cooperate well when digital health devices are used on them?

o   Very Uncooperative

o   Somewhat Uncooperative

o   Neutral

o   Cooperative

o   Very Cooperative

Part B: Patients perceptions towards digital health services

Section 1: Socio-demographic details

1.1 Age in years:

1.2 Gender:

1.3 Marital status:

1.4 Educational status:

1.5 Occupational status:

1.6 Total family income? Rs. ___________________     

1.7 Type of family:

1.8 Address:

Section 2: Experience with digital vital monitoring

2.1 How would you describe your overall experience with digital health monitoring, if applicable?

o   Very Positive

o   Positive

o   Neutral

o   Negative

o   Very Negative

2.2 How comfortable do you feel with the use of digital health monitoring technology in your healthcare?

o   Very Comfortable

o   Comfortable

o   Neutral

o   Uncomfortable

o   Very Uncomfortable

2.3 How satisfied are you with the information and health education you received from digital health monitoring?

o   Very Satisfied

o   Satisfied

o   Neutral

o   Dissatisfied

o   Very Dissatisfied

2.4 In your opinion, what are the potential benefits of using digital vital monitoring for patient care?

o   Enhanced monitoring of health conditions

o   More convenience for patients

o   Improved communication with healthcare providers

o   Better understanding of your health status

2.5 Would you prefer to continue using digital vital monitoring in your healthcare visits or treatment in the future?

o   Yes

o   No

o   Unsure

Section 3:  

**Table 6:** Attitude of patients towards digital health devices

S.no	Variable	Strongly disagree	Disagree	Neutral	Agree	Strongly agree
1.	Technology has improved healthcare	1	2	3	4	5
2.	Health technologies are easy to use	1	2	3	4	5
3.	Healthcare providers rely too much on technology	1	2	3	4	5
4.	Real doctors and nurses cannot be replaced by technology	1	2	3	4	5
5.	Advocate for further integration of technology in the healthcare sector	1	2	3	4	5
6.	Health technology reduces human error	1	2	3	4	5
7.	The prospect of advancements in health technology is exciting	1	2	3	4	5
8.	Health technology is beneficial for everyone	1	2	3	4	5
9.	Concern that technology will not keep my health records private	1	2	3	4	5
